# Perceptions of Person-Centered Care Among Residents and Staff of Long-Term Care Communities

**DOI:** 10.1093/geroni/igaf122.553

**Published:** 2025-12-31

**Authors:** Elizabeth Seidel, Shih-Yin Lin, Tara Cortes

**Affiliations:** New York University Rory Meyers College of Nursing, New York, New York, United States; New York University Rory Meyers College of Nursing, New York, New York, United States; New York University/HIGN, New York, New York, United States

## Abstract

The convergence and divergence of perceptions of good person-centered care (PCC) practices as perceived by long-term care (LTC) residents and staff are under-explored. As part of the 2nd phase of a study to establish standards of care for PCC, 8 resident (n = 56) and 9 staff (n = 66) in-person focus groups were held at 5 LTC communities: The New Jewish Home (NY), Garden Spot Communities (PA) Bethel New Life (IL), Beatitudes Campus (AZ), and Sequoia Living (CA). Deductive content analysis was used to validate 7 PCC domains previously established by the University of Maine. All 7 PCC domains were mentioned in at least 50% of focus groups. While residents and staff agreed on certain aspects of PCC there were areas where there was a disconnect either in what was important in these domains, or the importance of the domains as demonstrated by how often these themes appeared in these focus groups. Staff Empowerment was the only domain that was discussed more often among staff groups and was a top domain from staff’s perspective. Resident Care, Choice, and Empowerment and Family Engagement were essential PCC domains agreed by all resident and staff focus groups, yet there were subtle differences between residents’ and staff’s framing of how to best address these domains. In order to better align expectations of recipient and delivery of PCC, it is important to compare and contrast perspectives of residents and staff. These findings also highlight the importance of staff empowerment in LTC staff’s ability to provide PCC.

